# Comprehensive Genomic Dataset of Chinese Lizardtail Herb and Comparative Genomic Analysis Provide Insights Into Its Paleo‐Polyploidization Event

**DOI:** 10.1002/ece3.70425

**Published:** 2025-03-30

**Authors:** Shunhui Cai, Chengyi Tang

**Affiliations:** ^1^ Nanjing University Nanjing China; ^2^ Yixing Genome Biotechnology Corporation Yixing China

**Keywords:** genomic dataset, paleo‐tetraploid, *PEL* gene family, *Saururus chinensis*

## Abstract

The Chinese lizardtail herb, *Saururus chinensis*, holds a prominent position in traditional Chinese medicine. In this study, we present a comprehensive genomic dataset for *S. chinensis*. Furthermore, comparative genomic analysis indicates that the extant genome of *S. chinensis* retains extensive traces of a paleo‐tetraploidization event. These traces are observable at both the macroscopic level of chromosomes and the microscopic level within specific gene families, such as the *PEL* (pseudo‐etiolation in light) gene family. Additionally, our findings further suggest that this paleo‐tetraploidization event drives an expansion of the *PEL* gene family in the *S. chinensis* genome, potentially facilitating its neo‐ and sub‐functionalization, and thereby contributing to the evolutionary adaptability of this species.

## Graphical abstract

In this study, we provided a comprehensive genomic dataset for *S. chinensis*. Additionally, we observed that the *S*. *chinensis* genome underwent a paleo‐tetraploidization event, followed by several chromosome fusion events, resulting in an aneuploid paleo‐tetraploid state in its current genomic structure. Furthermore, we also observed that this paleo‐tetraploidization event facilitated an expansion of the PEL family within the *S. chinensis* genome.

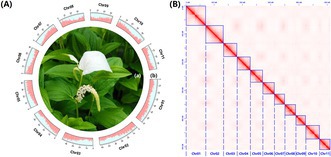

## 1 Introduction

**FIGURE 1:**
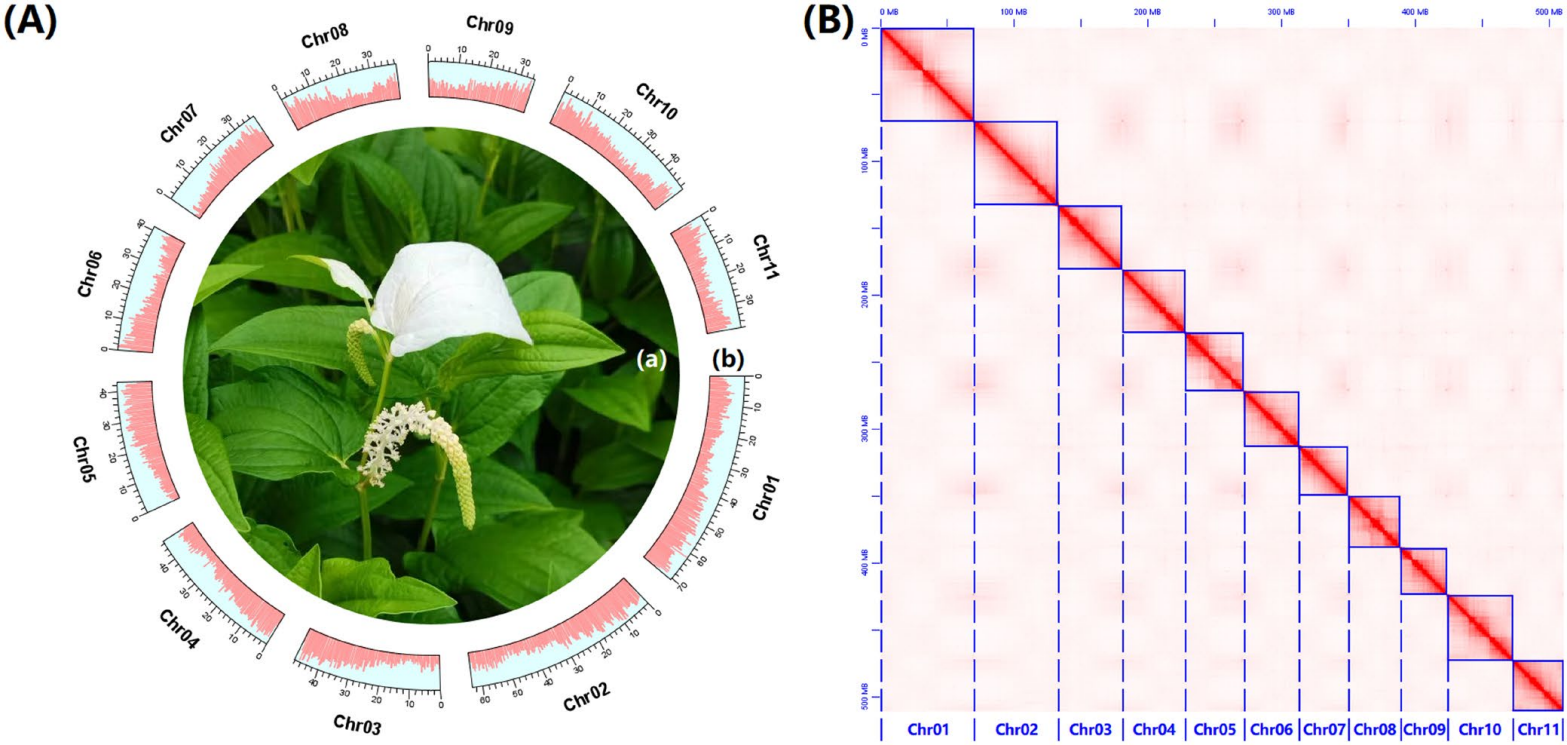
Overview of the newly sequenced *S. chinensis* genome (Version 4 in Table 2) in this study. (A) The newly sequenced *S. chinensis* (a) and its genic distribution in chromosomes (b); (B) Chromosome interaction signals in the newly sequenced *S. chinensis* genome (1*n* = 11).

The Chinese lizardtail herb (*Saururus chinensis*) (Figure 1A), also commonly known as “Sanbaicao” within the scope of traditional Chinese medicine, is not only a well‐known traditional herb used as a treatment for conditions such as edema, asthma, jaundice, gonorrhea and various other ailments, but it is also a core species within the family Sauraceae, in the order Piperales (Liu et al. 2020). Currently, the Piperales is divided into three families, namely Aristolochiaceae, Piperaceae, and Saururaceae (The Angiosperm Phylogeny Group 2016). It is interesting to note that the two perianth‐less (lacking petals and/or sepals) families, Piperaceae and Saururaceae, exhibit marked dissimilarity when juxtaposed with the perianth‐bearing family Aristolochiaceae (Jaramillo, Manos, and Zimmer 2004; Remizowa, Rudall, and Sokoloff 2005). Due to its perianth‐less floral composition and easy artificial propagation, *S. chinensis* has primarily found utility in genetic investigations to understand the origin of primitive flowering plants (Zhao et al. 2013; Zhao, Zhang, and Li 2021; Xue et al. 2023).

To date, three versions of the *S. chinensis* genome have been published. However, the gene‐set completeness in these versions remains suboptimal, not only being significantly lower than the corresponding genome completeness, but also falling below 95% (Table 1). Moreover, Luo et al. (2024) have yet to release the chromosome‐level gene‐set annotation reported in their study (Table 1). Therefore, we emphasize the necessity of developing a high‐quality genomic dataset for *S. chinensis*, particularly including reliable gene‐set annotations. Such a resource would be valuable for comparative genomic analyses and related research endeavors.

**TABLE 1:** Three published versions of *S. chinensis* genome from previous studies.

Statistical types	Version 1 (Xue et al. 2023)		Version 2 (Luo et al. 2024)		Version 3 (Luo et al. 2024)	
	Scaffolds	Contigs	Scaffolds	Contigs	Scaffolds	Contigs
Total sequences	38	842	75	149	11	117
Total lengths (Mb)	539.018	538.938	533.607	533.606	516.470	516.408
N_50_ lengths (Mb)	47.843	1.429	14.961	10.524	48.440	10.471
Assembly level	Chromosome		Scaffold		Chromosome	
BUSCO evaluation (embryophyta_odb10, 1614) for genome assembly	**Complete: 1574 (97.52%)** [single‐copy: 1522 (94.30%), duplicated: 52 (3.22%)]; fragmented: 9 (0.56%); missing: 31 (1.92%)		**Complete: 1565 (96.96%)** [single‐copy: 1517 (93.99%), duplicated: 48 (2.97%)]; fragmented: 19 (1.18%); missing: 30 (1.86%)		**Complete: 1565 (96.96%)** [single‐copy: 1522 (94.30%), duplicated: 43 (2.66%)]; fragmented: 14 (0.87%); missing: 35 (2.17%)	
Download links for genome assembly	https://bioinformatics.psb.ugent.be/gdb/Saururus		https://www.ncbi.nlm.nih.gov/datasets/genome/GCA_037126625.1		https://www.ncbi.nlm.nih.gov/datasets/genome/GCA_035235625.1	
Total original gene models	36,140		20,561		N/A	
BUSCO evaluation (embryophyta_odb10, 1614) for original gene‐set	**Complete: 1474 (91.32%)** (single‐copy: 1407 [87.17%], duplicated: 67 [4.15%]); fragmented: 85 [5.27%]; missing: 55 [3.41%]		**Complete: 1515 (93.87%)** (single‐copy: 1465 [90.77%], duplicated: 50 [3.10%]); fragmented: 36 [2.23%]; missing: 63 [3.90%]		N/A	
Download links for original gene‐set	https://bioinformatics.psb.ugent.be/gdb/Saururus		https://doi.org/10.6084/m9.figshare.23735505.v1		N/A	
Total reannotated gene models	32,813		37,076		35,669	
BUSCO evaluation (embryophyta_odb10, 1614) for reannotated gene‐set	**Complete: 1577 (97.71%)** (single‐copy: 1506 [93.31%], duplicated: 71 [4.40%]); fragmented: 22 (1.36%); missing: 15 (0.93%)		**Complete: 1576 (97.64%)** (single‐copy: 1523 [94.36%], duplicated: 53 [3.28%]); fragmented: 19 (1.18%); missing: 19 (1.18%)		**Complete: 1576 (97.64%)** (single‐copy: 1531 [94.85%], duplicated: 45 [2.79%]); fragmented: 20 (1.24%); missing: 18 (1.12%)	
Download links for reannotated gene‐set	https://doi.org/10.6084/m9.figshare.27020335.v1		https://doi.org/10.6084/m9.figshare.27020335.v1		https://doi.org/10.6084/m9.figshare.27020335.v1	

Paleo‐polyploidization events, also known as whole‐genome duplication (WGD) events, are well established as pivotal in the evolutionary process of angiosperms (Jiao et al. 2011). These events are recognized as significant drivers of evolutionary adaptation and species diversification (Ren et al. 2018; Wu, Han, and Jiao 2020). However, the processes of post‐polyploid genome evolution are not uniform across all paleo‐polyploid species (Li et al. 2021). With advancements in long‐read sequencing technology, the availability of high‐quality chromosome‐level genomes has facilitated in‐depth investigations into plant genome evolution following paleo‐polyploidization events. A previous study (Xue et al. 2023) identified a paleo‐polyploidization event in the *S. chinensis* genome; however, the specific characteristics of this event and the subsequent genomic structure evolution remain unclear.

More interestingly, *S. chinensis* possesses prominent white bracts located beneath its inflorescences, playing a crucial role in insect pollination (Song et al. 2018). Xue et al. (2023) have identified a specific *PEL* (pseudo‐etiolation in light) gene in *S. chinensis*, designated as *ScPEL* (i.e., *Sc004_1478.1*), which inhibits chlorophyll biosynthesis and thereby contributes to bract whitening. However, the understanding of the *PEL* gene family in the *S. chinensis* genome, as well as its evolutionary process associated with the paleo‐polyploidization event, remains insufficiently elucidated.

In the present study, we provided a reliable and comprehensive genomic dataset for *S. chinensis*. Furthermore, we investigated the paleo‐polyploidization event in the *S. chinensis* genome from both macro chromosomal and micro gene‐familial perspectives. Additionally, we explored the relationship between this paleo‐polyploidization event and the *PEL* gene family. Our genomic dataset and findings are anticipated to provide valuable resources and insights for comparative genomics and molecular evolution research.

## 2 Materials and Methods

### 2.1 Plant Materials

Initially, some Chinese lizardtail herb seedlings were procured from Shenzhen Yuanzhihui Company through the 1688 online trading platform (https://shop3i77d84502842.1688.com). Subsequently, these seedlings underwent cultivation within a greenhouse environment (Figure 1A), maintaining a temperature of ~25°C and a photoperiod of 14 h of light contrasted with 10 h of darkness. After approximately 60 days, fresh leaves obtained from a vigorous individual were collected for genome and Hi‐C sequencing. Simultaneously, fresh leaves and stems from the same individual were collected for transcriptome sequencing.

### 2.2 Genome, Hi‐C, and Transcriptome Sequencing

Total DNAs were extracted using the Magnetic Plant Genomic DNA Kit (Cat. no: 4992407; Tiangen, China). A paired‐end library with an insert size of 350 bp was constructed using the TIANSeq Fast DNA Library Kit (Cat. no: 4992261; Tiangen) and then sequenced using an Illumina NovaSeq6000 sequenator (Illumina, USA). An amplification‐free whole genome sequencing library was constructed using the Ligation Sequencing Kit (No: SQK‐LSK110; ONT, UK) and then sequenced by a PromethION sequenator (ONT). A Hi‐C library was constructed following a recognized Hi‐C protocol (restriction enzyme: HindIII) described in earlier studies (Grob, Schmid, and Grossniklaus 2014; Rao et al. 2014; Tang et al. 2020; Qin et al. 2021; Cui et al. 2022; Xue et al. 2023; Luo et al. 2024) and then sequenced by an Illumina NovaSeq6000 sequenator (Illumina). Total RNAs were extracted using an RNAprep Pure Plant Kit (Cat. no: 4992237; Tiangen). A cDNA library was constructed using the TIANSeq Fast RNA Library Prep Kit (Cat. no: 4992376; Tiangen) and then sequenced using an Illumina NovaSeq6000 sequenator (Illumina).

### 2.3 Data Processing

Fastp v0.23.2 (Chen et al. 2018) filtered Illumina raw data to remove adapters, low‐quality reads, and poly‐N reads. NanoFilt v2.8.0 (De Coster et al. 2018) filtered ONT raw data to remove too‐short reads (i.e., length < 2 kb) and low‐quality reads (i.e., RQ < 7.0).

### 2.4 Genome Size Estimation

The Illumina clean data (Table S1) was applied for genome size estimation. K‐mers were counted and exported to a histogram file using Jellyfish v2.3.0 (key parameters: jellyfish count ‐m 17, 19, or 21; jellyfish histo ‐h Max_count) (Marcais and Kingsford 2011). Preliminary genome sizes were calculated using GenomeScope 2.0 (Ranallo‐Benavidez, Jaron, and Schatz 2020) or GCE v1.0.2 (Liu et al. 2013), and the final genome size was averaged over the preliminary genome sizes.

### 2.5 Genome Assembly

The ONT clean data (Table S1) was used for genome assembly. First, NextDenovo v2.5.0 (key parameters: seed_depth = 999, nextgraph_options = ‐a 1 ‐u 1 ‐G) (Hu et al. 2023) was executed for contig‐level assembly. Over‐redundant contigs were removed via purge_dups v1.2.6 (key parameters: ‐a 50) (Guan et al. 2020). NextPolish v1.4.1 (key parameters: task = 551,212) (Hu et al. 2020) was then used for genome polishing based on ONT and Illumina clean data (Table S1). Subsequently, the Hi‐C clean data (Table S1) was mapped to the polished contig‐level assembly using Juicer v1.6 (key parameters: ‐s HindIII) (Durand et al. 2016) and ordered to a chromosome‐level assembly via 3D‐DNA (Dudchenko et al. 2017). Finally, Juicebox v1.11.08 (Durand et al. 2016) was used to manually curate this chromosome‐level assembly.

### 2.6 Genome Integration

Utilizing the chromosome‐level assembly provided by Luo et al. (2024) (Version 3 in Table 1) as a framework, we integrated genome assemblies from Xue et al. (2023) (Version 1 in Table 1) and this study (Version 4 in Table 2) via RagTag v2.1.0 (key parameters: ‐‐aligner minimap2) (Alonge et al. 2022). Subsequently, genome polishing for the merged assembly was performed using NextPolish v1.4.1 (key parameters: task = 1212) (Hu et al. 2020) with Illumina clean data (Table S1).

### 2.7 Genome Annotation

Repetitive sequences were annotated via RepeatMasker v4.1.4 (https://www.repeatmasker.org), based on a combined database including Dfam v3.7 (Storer et al. 2021) plus a de novo custom library of *S. chinensis* constructed via RepeatModeler v2.0.4 (key parameter: ‐LTRStruct) (Flynn et al. 2020). Subsequently, protein‐coding genes were annotated as the following process: (1) Repetitive sequences were masked first; (2) AUGUSTUS v3.5.0 (Stanke et al. 2008) and GeneMark‐EP+ v4.71 (Bruna, Lomsadze, and Borodovsky 2020) were used for ab initio predictions; (3) Exonerate v2.4.0 (Slater and Birney 2005) was applied to homological predictions based on published genomes of two related species, that is, *A. fimbriata* (Qin et al. 2021) and *A. contorta* (Cui et al. 2022); (4) PASA v2.5.2 (Haas et al. 2003) was used to identify transcripts based on the transcriptome data (Table S1); (5) The total results were integrated into a joint gene‐set using Maker v3.01.03 (key parameters: softmask = 1; min_protein = 49) (https://www.yandell‐lab.org/software/maker.html).

### 2.8 Completeness Evaluation

The completeness of the genome and gene‐set was evaluated using BUSCO v5.2.2 (key parameters: ‐‐augustus ‐l embryophyta_odb10 ‐m genome or proteins) (Manni et al. 2021).

### 2.9 Comparative Genome Analysis

Gene‐sets were employed for sequence similarity search via BLASTP 2.13.0+ (key parameter: ‐evalue 1e‐5 ‐max_target_seqs 5) (Camacho et al. 2009). Subsequently, the obtained results were further analyzed using MCScanX (Wang et al. 2012) to identify collinearity within monophyletic or crossed species. In addition, all gene pairs within the identified collinear blocks were aligned individually via MUSCLE v3.8.31 (Edgar 2004), predicated on their amino acid sequences, and all alignments were then transformed back to nucleotide sequences. The computation of Ks values for individual gene pairs was performed via KaKs_Calculator 3.0 (Zhang 2022), based on these nucleotide alignments. The peaks in the Ks distribution curves of monophyletic species were indicative of polyploidization events, while the peaks in the Ks distribution curves of crossed species were indicative of divergence events.

### 2.10 Gene Family Analysis

Initially, the hmmscan module (key parameter: ‐E 1e‐05) within HMMER 3.3.2 (Potter et al. 2018) was employed for domain identification in the ScPEL protein (i.e., the Sc004_1478.1 protein from the Xue et al.'s (2023) original gene‐set, Table 1). Subsequently, utilizing the PF09713 domain as a query, domain similarity search was performed via the hmmsearch module (key parameter: ‐E 1e‐05) within HMMER 3.3.2 (Potter et al. 2018). Concurrently, utilizing the ScPEL protein as a query, sequence similarity search was performed via BLASTP 2.12.0+ (key parameters: ‐evalue 1e‐5 ‐max_target_seqs 500) (Camacho et al. 2009). The redundant PEL members were removed from the final output set. Following this, the identified PEL proteins were aligned via MUSCLE v3.8.31 (Edgar 2004). The initial alignment was trimmed via trimAl v1.4.1 (key parameter: ‐gt 0.50) (Capella‐Gutierrezy, Silla‐Martínez, and Gabaldon 2009). The trimmed alignment was used as the basis for constructing a phylogenetic tree via IQ‐TREE v2.2.2 (best‐fit model: JTT + I + G4; key parameters: ‐‐seqtype AA ‐m MFP ‐‐alrt 1000 ‐B 1000) (Minh et al. 2020) according to the ML (maximum likelihood) method. The delineation of the *PEL* gene family was based on the hierarchical structure of the phylogenetic tree. Additionally, the “duplicate_gene_classifier” module within MCScanX (Wang et al. 2012) was utilized to determine the duplication types for each *PEL* gene family members.

## 3 Results and Discussion

### 3.1 *Saururus chinensis* Genome Sequencing, Assembly, Integration, and Annotation

A dataset for the newly sequenced *S. chinensis* genome was generated, comprising ~22.95 Gb of Illumina reads, ~66.56 Gb of ONT reads, and ~40.50 Gb of Hi‐C reads (Table S1). The estimated genome size of *S. chinensis* was ~528.084 Mb (Table S2), consistent with the results of Xue et al. (2023) and Luo et al. (2024). The final assembled genome spanned ~522.246 Mb, with a Scaffold N_50_ length of ~46.947 Mb and a Contig N_50_ length of ~4.179 Mb (Version 4 in Table 2), and revealed the presence of 11 chromosomes (Figure 1B), consistent with previous studies (Okada 1986; Xue et al. 2023; Luo et al. 2024). Genome annotations indicated that ~275.770 Mb (~52.807% of the total genome) consisted of repetitive sequences, including ~211.582 Mb of interspersed repeats (~40.515%), ~4.120 Mb of tandem repeats (~0.788%), and ~58.793 Mb of unclassified repeats (~11.258%) (Table S3). Moreover, the genome contained a total of 32,124 protein‐coding gene models (Figure 1A and Table 2). BUSCO evaluation demonstrated that 95.54% and 95.91% of the complete BUSCOs were identified in the whole genome and total gene models, respectively (Table 2), indicating an acceptable level (i.e., ≥ 95%) of completeness for both the genome assembly and gene‐set annotations.

**TABLE 2:** Two new versions of *S. chinensis* genome from this study.

Statistical types	Version 4 (This study)		Version 5 (This study)	
	Scaffolds	Contigs	Scaffolds	Contigs
Total sequences	72	323	11	55
Total lengths (Mb)	522.247	522.222	515.801	515.797
N_50_ lengths (Mb)	46.948	4.180	48.296	40.347
Assembly level	Chromosome		Chromosome	
BUSCO evaluation (embryophyta_odb10, 1614) for genome assembly	**Complete: 1542 (95.54%)** (single‐copy: 1496 [92.69%], duplicated: 46 [2.85%]); fragmented: 13 (0.80%); missing: 59 (3.66%)		**Complete: 1577 (97.71%)** (single‐copy: 1528 [94.67%], duplicated: 49 [3.04%]); fragmented: 12 (0.74%); missing: 25 (1.55%)	
Download links for genome assembly	https://www.ncbi.nlm.nih.gov/datasets/genome/GCA_041464145.1		https://doi.org/10.6084/m9.figshare.27020347.v1	
Total gene models	32,124		31,774	
BUSCO evaluation (embryophyta_odb10, 1614) for gene‐set	**Complete: 1548 (95.91%)** (single‐copy: 1499 [92.87%], duplicated: 49 [3.04%]); fragmented: 18 (1.12%); missing: 48 (2.97%)		**Complete: 1579 (97.83%)** (single‐copy: 1533 [94.98%], duplicated: 46 [2.85%]); fragmented: 23 (1.43%); missing: 12 (0.74%)	
Download links for gene‐set	https://doi.org/10.6084/m9.figshare.25035707.v1		https://doi.org/10.6084/m9.figshare.27020347.v1	

We also reannotated the gene models in the three published versions of the *S. chinensis* genome. The reannotation results identified 32,812, 37,076, and 35,669 protein‐coding gene models, respectively, in these genomes (Table 1). BUSCO evaluation demonstrated that 97.71%, 97.64%, and 97.64% of complete BUSCOs were detected in the respective reannotated gene‐sets (Table 1). These results reflected that a significant improvement in completeness compared to the original gene‐set annotations (Table 1) and confirmed that the original annotations were indeed suboptimal.

Furthermore, we integrated the two previously published chromosome‐level assemblies (i.e., Versions 1 and 3 in Table 1) with the assembly generated in this study (i.e., Version 4 in Table 2), culminating in a merged genome assembly (i.e., Version 5 in Table 2). This merged version exhibited a notable Contig N50 length of ~40.374 Mb and contained a total of 31,774 protein‐coding gene models (Table 2). BUSCO evaluation revealed that 97.71% of complete BUSCOs were identified in the genome assembly, while 97.83% were detected in the gene‐set annotations (Table 2). These results indicated that the merged assembly has outperformed all individual assemblies in terms of both contiguity and completeness.

In summary, we have successfully produced a comprehensive genomic dataset for *S. chinensis*, featuring highly contiguous genome assemblies and robust gene‐set annotations. We are confident that this genomic resource will contribute to comparative genomics.

### 3.2 *Saururus chinensis* Genome Is an Aneuploid Paleo‐Tetraploid

**FIGURE 2:**
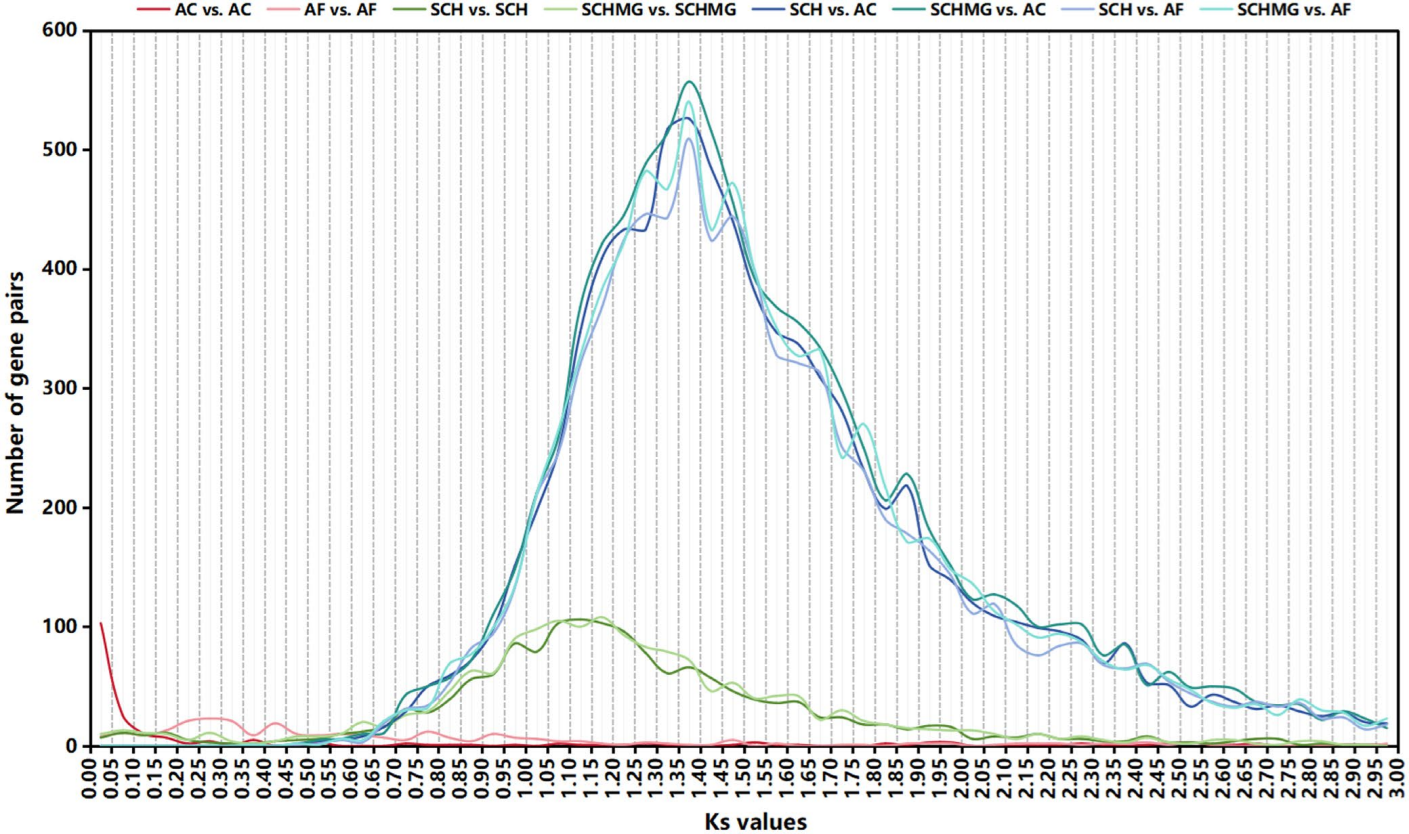
Ks distribution curves. AC, *A. contorta* genome (Cui et al. 2022); AF, *A. fimbriata* genome (Qin et al. 2021); SCH, newly sequenced *S. chinensis* genome (Version 4 in Table 2); SCHMG, merged *S. chinensis* genome (Version 5 in Table 2).

**FIGURE 3:**
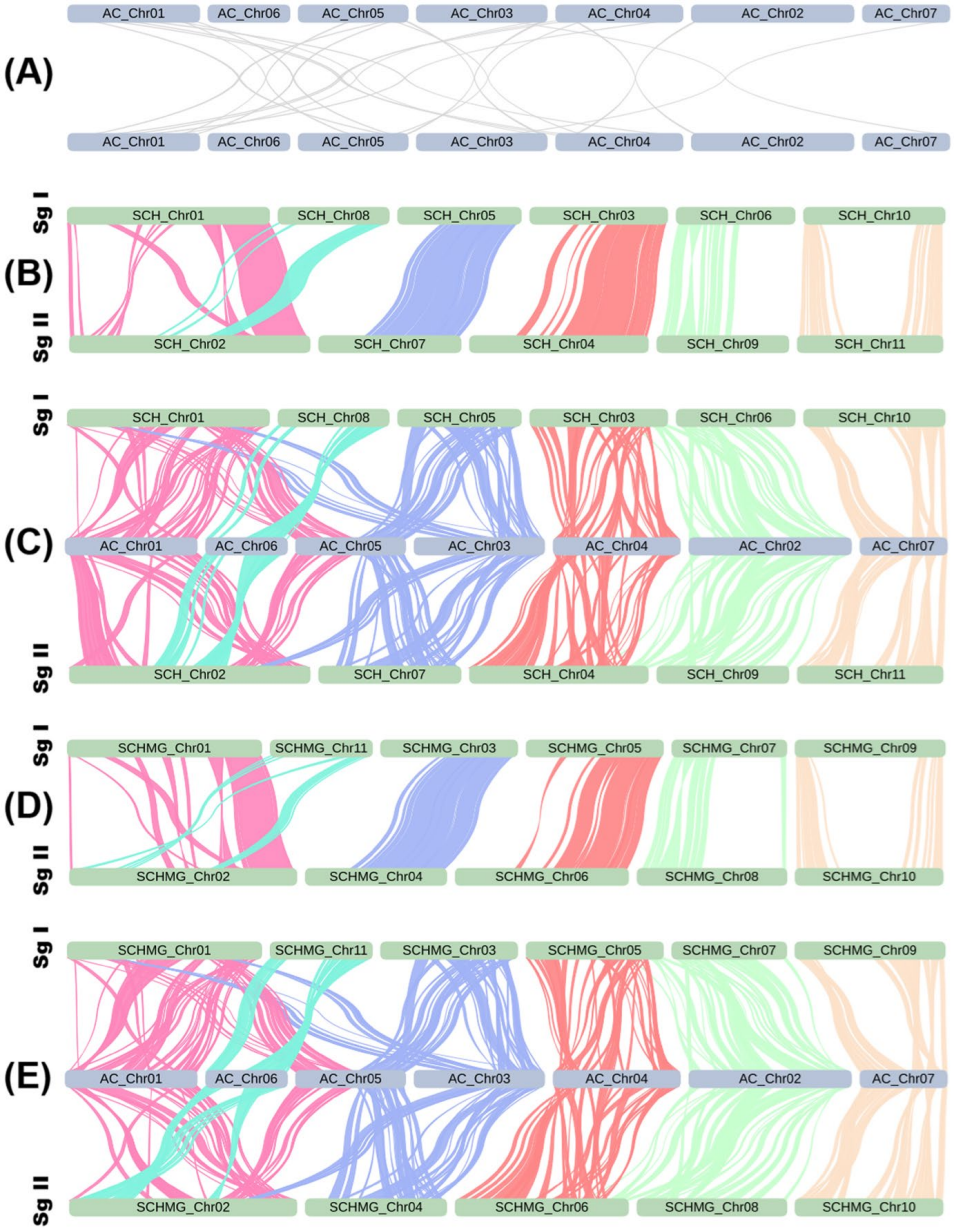
Chromosomal collinearity. (A) *A. contorta* genome (Cui et al. 2022); (B) Newly sequenced *S. chinensis* genome (Version 4 in Table 2); (C) *A. contorta* genome versus newly sequenced *S. chinensis* genome (Version 4 in Table 2); (D) Merged *S. chinensis* genome (Version 5 in Table 2); (E) *A. contorta* genome versus merged *S. chinensis* genome (Version 5 in Table 2). Sg I and Sg II, subgenome I and Subgenome II in the *S. chinensis* genomes.

The Ks distribution curves indicated that Ks_(SCH_vs_AC)_ ≈ Ks_(SCH_vs_AF)_ ≈ Ks_(SCHMG_vs_AC)_ ≈ Ks_(SCHMG_vs_AF)_ ≈ 1.375 is significantly higher than Ks_(SCH_vs_SCH)_ ≈ Ks_(SCHMG_vs_SCHMG)_ ≈ 1.150 (Figure 2), which suggested that *S. chinensis* underwent a paleo‐polyploidization event following its divergence from the two *Aristolochia* species. Moreover, two versions of the *S. chinensis* genome (i.e., Versions 4 and 5 in Table 2) are clearly organized into two subgenomes (Figure 3), further elucidating that this event manifested itself as a tetraploidization event. In addition, previous studies had highlighted that two *Aristolochia* species were typically diploid and had not undergone a polyploidization event after the ancestral ε event (Jiao et al. 2011; Qin et al. 2021; Cui et al. 2022). Our results also supported this proposition, which was evident at both the Ks distribution and genome collinearity levels (Figures 2 and 3). Consequently, it became apparent that *S. chinensis*, after the tetraploidization event, still retained abundant vestiges of genome duplication from that epoch, and that it had not been thoroughly re‐diploidized even to the present day. In other words, at the macroscopic chromosome level, unlike the typical diploid (e.g., two *Aristolochia* species), the *S. chinensis* genome could be referred to as a “paleo‐tetraploid.” Furthermore, the *S. chinensis* genome comprises 11 chromosomes, an odd number that precludes division into two balanced subgenomes. This anomaly results from several genome fusions that occurred following the paleo‐tetraploidization event within the *S. chinensis* genome. A prominent illustration is the chromosome‐level fusion of the collinear blocks corresponding to the existing Chromosome SCH_Chr08 (or SCHMG_Chr11) into the existing Chromosome SCH_Chr02 (or SCHMG_Chr02) (Figure 3). Consequently, a more precise characterization of the *S. chinensis* genome would be as an “aneuploid paleo‐tetraploid.”

### 3.3 Paleo‐Tetraploidization Promotes PEL Family Expansion in the *S. chinensis* Genome

**FIGURE 4:**
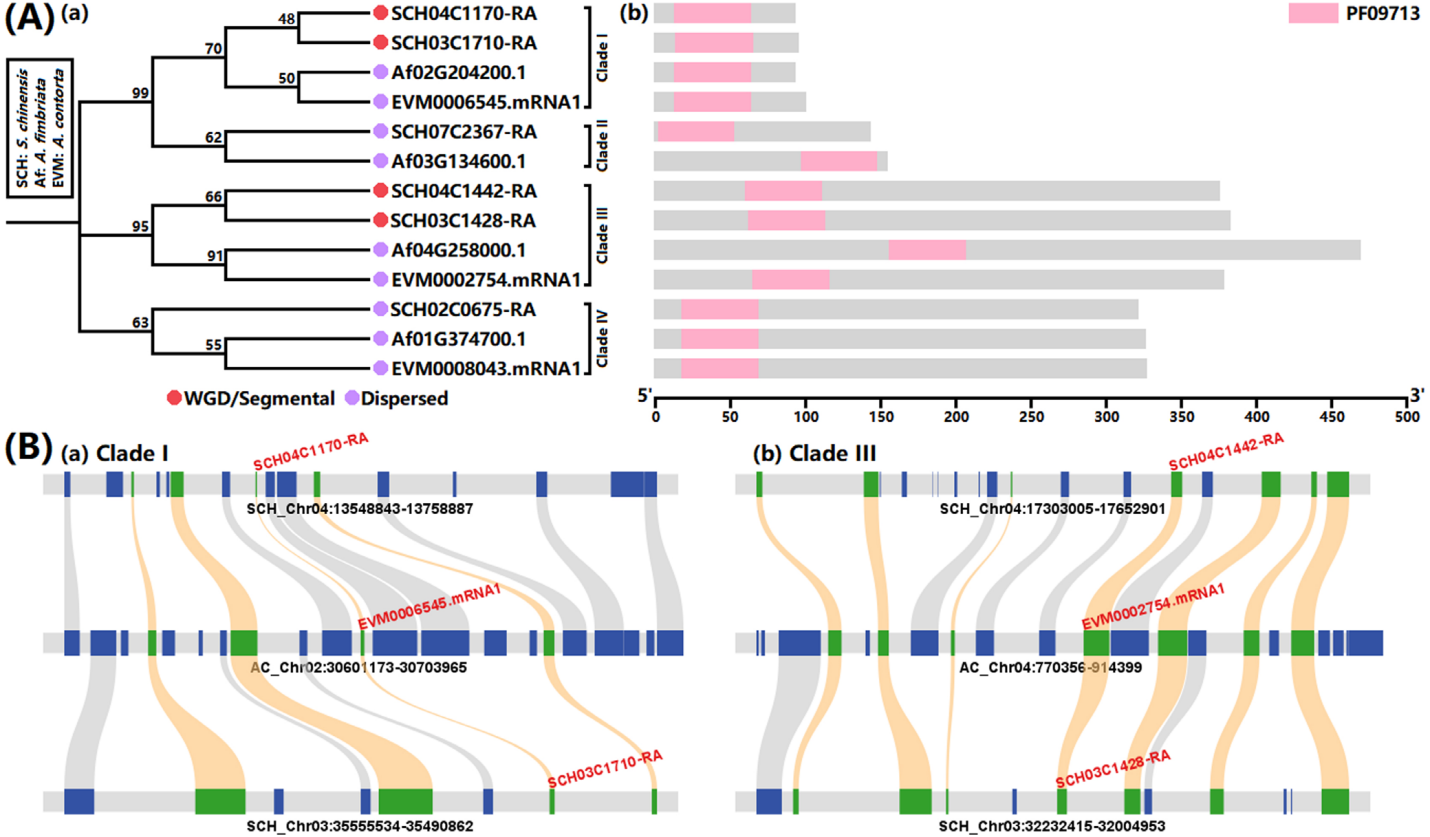
PEL family and its evolutionary process. (A) Phylogenetic structure (a) and sequence characteristics (b) of PEL family; (B) Microscopic collinearity of PEL family expansion (a. Clade I and b. Clade III) between *A. contorta* (AC) with *S. chinensis* (SCH; Version 4 in Table 2).

We discerned a singular complete domain, namely “PF09713|A_thal_3526” (https://www.ebi.ac.uk/interpro/entry/pfam/PF09713/), within the ScPEL protein, indicating its distinctive structural feature (Table S4). Utilizing domain plus sequence similarity searches, we found that the ScPEL (i.e., Sc004_1478.1) protein from Xue et al.'s original gene‐set annotation is equivalent to the SCH04C1170‐RA protein in our Version 4 annotation (Table S5). Furthermore, we identified a total of six PEL members in the *S. chinensis* genome (Version 4 in Table 2), four in the *A. fimbriata* genome, and three in the *A. contorta* genome (Figure 4A and Tables S4 and S5). A phylogenetic analysis delineated the PEL family into four distinct clades (Figure 4A). There also were discernible differences in the length of amino acid sequences corresponding to the four clades, with Clade III exhibiting the highest length, followed by Clade IV, Clade II, and Clade I (Figure 4A). Interestingly, a quantitative expansion of the *PEL* genes was observed in *S. chinensis* compared to two *Aristolochia* species (i.e., 6:4:3). This genic expansion was attributed to the paleo‐tetraploidization event that occurred within the *S. chinensis* genome (Figures 2 and 3), resulting in gene duplication in Clades I and III (Figure 4A), and also revealing the enduring imprint of the paleo‐tetraploidization event on the genic landscape at the microscopic scale (Figure 4B). This expansion augmented genic resources, potentially contributing to the neo‐ and sub‐functionalization of the *PEL* gene family members within the *S. chinensis*, and thus to the evolutionary origin of the distinctive white bracts.

## 4 Conclusion

In this study, we provided a comprehensive genomic dataset for *S. chinensis*, comprising highly contiguous genomes and well‐curated gene‐set annotations. Our analyses revealed that the *S. chinensis* genome underwent a paleo‐tetraploidization event, followed by several chromosomal fusion events, resulting in an “aneuploid paleo‐tetraploid” configuration in its current genomic structure. Moreover, we identified that this paleo‐tetraploidization event drives an expansion of the *PEL* gene family within the *S. chinensis* genome. We propose that this expansion played a crucial role in the neo‐ and sub‐functionalization of *PEL* gene family members, ultimately contributing to the evolutionary origin of white bracts in *S. chinensis*.

## Author Contributions

**Shunhui Cai:** data curation (supporting), investigation (supporting), methodology (supporting), software (supporting), visualization (equal), writing – original draft (supporting), writing – review and editing (equal). **Chengyi Tang:** conceptualization (lead), data curation (lead), formal analysis (lead), funding acquisition (lead), investigation (lead), methodology (lead), project administration (lead), resources (lead), software (lead), supervision (lead), visualization (equal), writing – original draft (lead), writing – review and editing (equal).

## Conflicts of Interest

The authors declare no conflicts of interest.

### Open Research Badges

This article has earned an Open Data badge for making publicly available the digitally‐shareable data necessary to reproduce the reported results. The data is available at https://www.ncbi.nlm.nih.gov/bioproject/PRJNA1066966; https://doi.org/10.6084/m9.figshare.25035707.v1; https://doi.org/10.6084/m9.figshare.27020335.v1; https://doi.org/10.6084/m9.figshare.27020347.v1.

## Supporting information


Tables S1‐5.

## Data Availability

All sequencing data generated in this study have been deposited in the NCBI database under accession number PRJNA1066966, with corresponding SRA IDs detailed in Table S1. Additionally, the assembled genome sequences and their associated gene‐set annotations have been made publicly accessible via the NCBI and/or Figshare database (Tables 1 and 2).
